# A case of alternating hemiplegia in 2-month-old children with nystagmus as the first symptom: A case report

**DOI:** 10.1097/MD.0000000000039774

**Published:** 2024-09-27

**Authors:** Qicheng Qiao, Qiubo Li

**Affiliations:** a School of Clinical Medicine, Jining Medical University, Jining, China; b Department of Paediatrics, Affiliated Hospital of Jining Medical University, Jining, China.

**Keywords:** alternating hemiplegia in children, ATP1A3 gene, epilepsy, nystagmus

## Abstract

**Rationale:**

This case report delves into the rare neurological condition known as alternating hemiplegia of childhood (AHC), focusing on its clinical manifestations, diagnostic approaches, and treatment options. AHC typically presents in infants under the age of 18 months with intermittent episodes of hemiplegia, often triggered by stressors such as environmental changes, bathing, or emotional stress. Recognizing the clinical features of AHC is crucial for early identification and intervention.

**Patient concerns:**

The paper presents a case of a 2-month-old child with nystagmus as the initial symptom, followed by limb movement disorder in the left upper limb and weakness in the right limbs. The child's condition did not improve with treatment at an external hospital, highlighting the complexity of the disease and the need for specialized care.

**Diagnoses:**

After a comprehensive review of the patient's medical history, physical examination, and imaging studies, the child was diagnosed with AHC. The diagnosis was confirmed through video electroencephalogram and whole-exome gene detection, which revealed a de novo mutation in the ATP1A3 gene, identified as pathogenic according to the American College of Medical Genetics and Genomics guidelines.

**Interventions:**

The child was admitted to Peking University First Hospital and treated with levetiracetam and flunarizine oral administration. These medications were chosen for their efficacy in managing the symptoms of AHC, particularly the hemiplegic episodes.

**Outcomes:**

Post-treatment, the child experienced a reduction in the frequency and intensity of hemiplegic attacks compared to the initial stage. However, the child still exhibited paroxysmal symptoms and abnormal eye movements, and developmental milestones were delayed, indicating the need for ongoing care and monitoring.

**Lessons:**

This case underscores the importance of early recognition and prompt intervention in managing children with AHC. The varied clinical presentations of AHC necessitate vigilance for early differential diagnosis. Although AHC is currently incurable, appropriate treatment can mitigate the impact of complications and improve the long-term quality of life for affected children, facilitating better societal integration.

## 1. Introduction

Alternating hemiplegia of childhood (AHC) is a rare neurological condition that typically presents in infants under the age of 18 months with intermittent episodes of hemiplegia, affecting one or both sides of the body. These episodes are often triggered by stressors such as environmental changes, bathing, or emotional stress. They can manifest alone or alongside other symptoms including autonomic instability, altered consciousness, and movement disorders, such as dystonia, ataxia, and choreoathetosis. Patients may also develop developmental or intellectual disabilities and experience epileptic seizures. Notably, certain symptoms appear in distinct phases and typically resolve during sleep. The incidence of AHC is estimated at 1 in 1,000,000 among children under the age of 16 years, a figure that may underrepresent the true prevalence due to the condition’s diverse clinical presentations and the limited genetic testing in past studies.^[1,2]^ The precise pathophysiology of AHC, including its associated comorbidities, remains incompletely understood. However, recent advances in molecular genetics have led to a clearer understanding of the genes implicated in AHC, facilitating earlier and more definitive diagnoses. The most commonly associated genes are mutations in ATP1A2 and ATP1A3, which are responsible for producing different alpha subunits of the neuronal Na+-K + ATPase pump. Individuals with ATP1A3 mutations are more frequently affected than those with ATP1A2 mutations. The increased application of genetic testing has not only improved the differentiation of AHC from other similar disorders but also expanded the recognized clinical spectrum of the condition. This article reports a case of alternating hemiplegia in children with nystagmus as the first symptom.

## 2. Case report

**Figure 1.**
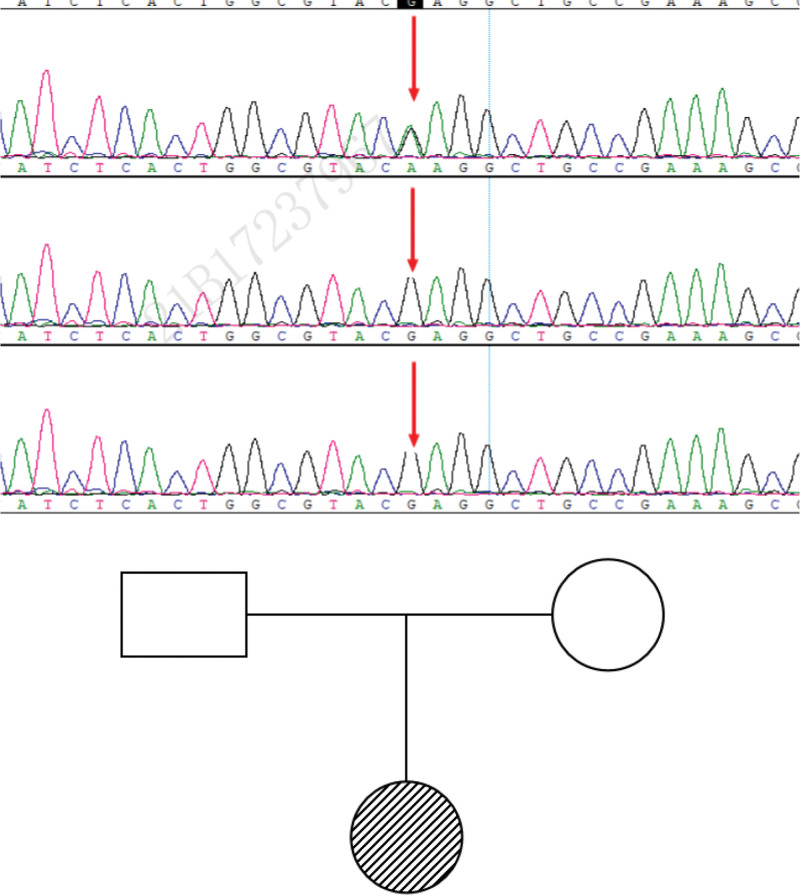
Total explicit gene test.

The female patient, 1 year and 7 months of age, developed ocular fibrillation 2 months after birth, limb movement disorder of the left upper limb 6 months after birth, and right limb weakness 3 days after birth, lasting from 3 to 6 days. The patient was treated with lysine B12 oral solution in an external hospital, but the condition did not relieve, fever appeared at 7 months after birth, and convulsions occurred once when the body temperature was normal 5 days later. It was manifested as the pale face, stiffness, and shaking of the limbs, which lasted for about half an hour and then relieved. The child is the first birth, full-term natural birth, no abnormal birth history, no history of hypoxia and asphyxia after birth, no special family history, physical examination, can sit, there are independent grasp, delivery, can laugh, double pupils and another large circle, sensitive to light reflex, no obvious positive signs in the heart, lungs, abdomen, pathological signs and meningeal stimulation signs did not lead; laboratory examination: blood routine, liver and kidney function, myocardial enzymes, blood biochemistry, blood lactic acid, hematuria screen, plasma ammonia, etc, were not abnormal. Improved magnetic resonance imaging showed that the extracerebral space was widened, and no abnormality remained; video electroencephalogram (EEG) showed abnormal EEG, no obvious epileptic discharge, irregular slow wave emission can be seen in the left temporal region, and the amplitude is higher than that on the right side. Perfect whole exon gene detection indicates ATP1A3 neonatal heterozygous mutation. The mutation site was NM_152296.4:c.2243G > A (P.Lu815Lys; Fig. 1). It was a pathogenic mutation according to ACMG guidelines, and the pathogenic evidence was PS2_Very Strong + PSPM2 + PP2 + PP3 + PP4. On January 39, 2023, he was admitted to the First Hospital of Peking University. Since then, he has been taking oral administration of levetiracetam and flunarizine. So far, he has not twitched, and the attack has improved compared with before, but he still has paroxysmal symptoms, abnormal eye movement, the outward inclination of both eyes to one side, paroxysmal limb movement disorder posture, once in 1 to 2 weeks, hemiplegia once in about 1 week, and physical examination: at present will shout “father, mother” and other blind speech, speech expression is not fluent, not clear, the completion of large movements is not good, can grasp, fine movements are not good, cannot stand, support and stand unstable, and the development is slower than that of children of the same age.

## 3. Discussion

AHC is a rare neurological disorder characterized by paroxysmal transient hemiplegic events involving one or both sides of the body, usually occurring before the child is 18 months old. Paroxysmal episodes may occur independently or may be associated with other clinical manifestations, such as autonomic dysfunction, altered consciousness, and abnormal movements, such as limb movement disorder, ataxia, and dance athetosis, and the child may potentially exhibit developmental delays, intellectual impairment, and seizures^[3,4]^

The current diagnostic criteria are the diagnostic criteria formulated by Heinzen et al^[5]^: the age of onset is before 18 months; recurrent hemiplegia, involving both sides of the body in at least some episodes; other paroxysmal disorders, including tonic (tonic episodes), nystagmus, strabismus, dyspnea, and other autonomic neurological phenomena that occur during or alone with hemiplegic episodes; episodes of bilateral hemiplegia or quadriplegia that begin with a generalization of the episode of hemiplegia or begin with the episode of bilateral hemiplegia; the symptoms disappear immediately after falling asleep and relapse 10 to 20 minutes after waking up, with a longer duration; and evidence of developmental delays and neurological abnormalities, including dance athetosis, limb movement disorder, or ataxia. Mikati et al^[1]^ adjusted for this as follows: hemiplegic episodes alternate between one side and the other; episodes of movement disorders, such as limb movement disorder, dance athetosis, ataxia, and intermittent abnormalities of eye movement; and may also include developmental and cognitive delays with or without spastic diplegia, spastic quadriplegia, or hypotonia. In this case, the child initially presented with nystagmus as an early symptom not abnormalities in limb movement, thus highlighting the need to be vigilant that children with AHC may exhibit a variety of complications as their initial symptoms. This is beneficial for early differential diagnosis.

Symptoms observed in AHC can sometimes be confused with those of other conditions, both serious and less severe. To distinguish AHC, it is crucial to rule out conditions such as moyamoya angiopathy and various mitochondrial disorders, including Kearns-Sayre syndrome and mitochondrial encephalomyopathy, lactic acidosis, and stroke-like episodes. Moyamoya angiopathy, a vascular issue, involves the narrowing of the internal carotid arteries’ terminal sections and the emergence of an abnormal collateral circulation network, potentially leading to reduced cerebral blood flow, brain ischemia in children, and strokes in adults. This condition might manifest with transient ischemic attacks, visual problems, speech difficulties, and unilateral weakness or paralysis that could be mistaken for AHC episodes. Kearns-Sayre syndrome, a form of chronic progressive external ophthalmoplegia, is characterized by the onset before the age of 20 years, chronic progressive external ophthalmoplegia, and retinopathy with pigmentary degeneration. Patients may exhibit additional symptoms such as heart block, elevated cerebrospinal fluid protein levels, ataxia, deafness, cognitive delays, and hormonal imbalances.^[6]^ Mitochondrial encephalomyopathy, lactic acidosis, and stroke-like episodes typically appear in children or young adults with recurring episodes of encephalopathy, myopathy, headaches, and progressive focal neurological deficits.^[7]^ When differentiating AHC, it is also important to consider other ATP1A3-related disorders, such as early-onset infantile epileptic encephalopathy and rapid-onset dystonia parkinsonism. Benign familial nocturnal alternating hemiplegia of childhood, which may be associated with migraines, presents with recurrent hemiplegic episodes during sleep in children without neurological or cognitive deficits.^[8]^

The largest number of cases studied so far was by Panagiotakaki et al^[4]^ in a large cohort of 157 patients with a median age of 20 months. All patients experienced an episode of hemiplegia; 92% developed cognitive impairment. The severity of symptoms remained stable, except for abnormal eye movement and hypotonia, until they disappeared in adulthood. Seven patients died as a result of severe paralytic episodes or seizures, 88% had limb movement disorder episodes, 86% had bilateral asthenia episodes, 72% had chorea and/or limb movement disorder, and 53% had seizures. Of the 103 patients performed by Sweney et al^[2]^ observed in the first 3 months of life, 100% were accompanied by varying degrees of cognitive involvement, 96% were accompanied by episodes of abnormal eye movement, and 50% were accompanied by nystagmus, which is most common in patients with nystagmus on one side. In addition, 43% of cases were accompanied by comorbidities including epilepsy, 83% had paroxysmal abnormal eye movement, and 56% had hemiplegic episodes at 6 months. Limb movement disorder symptoms preceded the onset of hemiplegia in 41% of cases, and the onset of limb movement disorder and paralysis occurred simultaneously in 35% of cases. The duration of both limb movement disorder and paralysis episodes varied from a few minutes to a few hours to even several days. Mikati et al^[1]^ also found that most patients with AHC were accompanied by comorbidities of varying degrees, such as neuropsychological abnormalities, developmental delays, seizures, motor dysfunction, migraines, and sleep disorders. Uchitel et al^[9]^ believe that seizures can be the earliest symptom, epilepsy can be locally related (focal) or systemic and can be accompanied by frequent episodes of nonepileptic decreased consciousness, epilepsy with AHC is usually drug-resistant but may respond to vagus nerve stimulation, and status epilepsia is common and usually refractory. Recurrent, most importantly, EEG epileptiform discharges often lag the onset of seizures. In this case, the EEG and brain magnetic resonance imaging of the child were not found abnormal, and the application of levetiracetam was well controlled, and no seizures occurred again. The current follow-up time is still short, the ocular abnormalities still exist, which do not appear as described above and can disappear with age, and the language and motor development of the child lag behind that of children of the same age.

The underlying pathophysiological mechanisms of AHC and various comorbidities are not fully understood, and molecular studies can provide a better understanding of the associated disease-causing genes. Mutations in ATP1A2 and ATP1A3 are the most common associated genes, and individuals with ATP1A3 mutations are more common than those with ATP1A2 mutations. More than half of all AHCs are caused by mutations in the ATP1A3 gene, which encodes 2 distinct α subunits of the neuron’s NA+-K + ATPase transmembrane ion pump, which is responsible in part for establishing and maintaining the electrochemical gradient of sodium and potassium ions across the neuron’s plasma membrane.^[10]^ The alpha 3 subunit is found primarily in the nervous system and is thought to be the most common form of the alpha subunit in the basal ganglia, hippocampus, and cerebellum.^[11]^ Despite the increasing number of pathogenic variants described, the most extensive cohort studies conducted in different populations indicate that the following 3 variants account for 60%, Asp801Asn variants for 30% to 43%, and p.Glu815Lys for 16% to 35%, in this case, such mutations.^[4,12]^

AHC is not yet curable and is generally treated acutely and seizure prevention, and acute phase treatment includes elimination of known triggers and avoidance of precipitating factors. Various drugs have been proposed to treat episodic seizures, but calcium channel blockers are currently the most effective, and the most common drug is flunarizine. In a study by Kossorotoff et al,^[6]^ of 44 patients with AHC treated with flunarizine, the frequency or severity of episodic seizures was clinically reduced in 27 patients (78%); hemiplegic episodes were not recurring in one patient, there was significant improvement in hemiplegic episodes in 2 patients, and only 4 patients had ineffective hemiplegic episodes. Episodes were significantly improved, and only 4 patients were ineffective. Pisciotta et al^[13]^ reported the use of flunarizine as a treatment, which provided good results in 50% of the patients with a reduction in the duration and frequency of episodes, and a reduction in the severity of episodes at the time of onset in 32% of the patients was more pronounced in the younger patients. In the study by Cordani et al,^[14]^ the effect of flunarizine appeared to be more effective than p.Asp801As in reducing the intensity and duration of seizures in patients with the p.Glu815Lys mutation. In this case, the child was treated with flunarizine, the hemiplegic seizure reoccurrence was significantly reduced in duration and intensity, and the interval between seizures was significantly prolonged. Patel et al^[15]^ retrospective review of available data of patients with AHC who received cannabidiol (CBD). Patients with AHC had a positive drug response to both drugs, flunarizine and CBD, with broadly similar efficacy, but in epileptic patients with seizures, CBD had a positive effect on epileptic patients, and flunarizine had a less pronounced effect. CBD was well tolerated with no patients discontinuing it due to side effects.

For this study, we can conclude that AHC is a complex and heterogeneous disease, with hemiplegia being just 1 of its manifestations. It primarily affects the autonomic nervous system, musculoskeletal system, and brain and is accompanied by seizures and cognitive deficits. Although AHC cannot be completely cured, there is currently no research, indicating that it limits the life expectancy of children. This is mainly because the complications of the disease can be fatal, such as aspiration. However, cognitive impairments and motor disabilities may persist over the long term. Through this case, the child’s early age of onset was observed, and early treatment has been very effective in controlling complications. The child has not had any further seizures, and the frequency of hemiplegic attacks has been significantly reduced compared to the initial stage. Reducing the impact of complications and improving the long-term quality of life for children with AHC, as well as better integration into society, are of utmost importance for children with AHC.

## Author contributions

**Conceptualization:** Qiubo Li.

**Data curation:** Qicheng Qiao.

**Writing – original draft:** Qicheng Qiao.

**Writing – review & editing:** Qiubo Li.
